# Therapeutic Notes

**Published:** 1896-11-21

**Authors:** 


### THERAPEUTIC NOTES.

### GELANTHUM.

This is the name of a new varnish prepared by Unna, and said by him to offer many advantages over his original dressing, "gelatum zincum." The latter preparation, as is well known, is a carefully made combination of oxide of zinc, gelatine, and glycerine, and has been extensively used for painting on eczematous surfaces, either with or without some other special medicament. The new varnish is a combination of tragacanth and gelatine, and its special merit is that it is capable of holding insoluble substances, such as oxide of zinc and sulphur, in a state of suspension; and other chemical substances can remain in this condition side by side without setting up chemical interactions or decompositions. The varnish cools very rapidly, and in so doing forms a smooth, dry, and even film, which can retain hydroscopic substances such as ichthyol without liquefaction, and without showing signs of undesirable dampness. It was found that the old gelatum zincum could not be employed with large percentages of salicylic acid, resorsin, and many other medicaments. With gelanthum they can be readily applied in large quantities, and without destroying the process. Fats and oleacious substances can also be combined to the extent of 10 per cent., and glycerine to the extent of 20 per cent., without delaying the rapid process of drying. The mode of preparation is as follows: Selected pieces of tragacanth are macerated with twenty volumes of water for four weeks in the cold, they are then steamed, and finally squeezed through muslin. The gelatine is soaked in cold water and filtered through Unna's steam funnel under pressure. The two products, the gelatine and the tragacanth, are then mixed in equal quantities, exposed to steam for two days, and then squeezed through muslin; 5 per cent. of glycerine is then added, and ·1 per cent. thymol. If any other medical principle has to be added which is insoluble in water, it must be made into a thin paste, and stirred into the mixture. A fatty substance must be made into an emulsion with gum. The advantages which are enumerated by Unna for this new varnish, as compared to other soluble varnishes, are as follows: (1) It is more easily painted on the surface; (2) it dries quicker and with smoother surface; (3) holding as it does a large quantity of water, it constitutes a cooler application; (4) it holds insoluble substances in suspension; (5) it allows of the use of hydroscopic substances, as well as fatty matters. As compared with gelatum zincum, Unna claims the following advantages: (1) It can be applied cold; (2) it requires no wool or other covering; (3) can be applied over short hair; (4) it forms a safe vehicle for all medicaments, and in large percentage. Over unguentum caseini it has similar advantages. This new preparation was exhibited by Unna at the Carlisle meeting of the B.M.A., and described by him in the *Deuch. Medic. Zeitung*, 1896, No. 73.

